# Multilayered regulation of cytoskeletal protein abundance: autoregulatory mechanisms of actin and tubulin

**DOI:** 10.1038/s12276-025-01615-w

**Published:** 2026-01-08

**Authors:** Yena Cho, Yong Kee Kim

**Affiliations:** 1 Muscle Physiome Research Center and Research Institute of Pharmaceutical Sciences, Sookmyung Women’s University, Seoul, Republic of Korea; 2 College of Pharmacy, Sookmyung Women’s University, Seoul, Republic of Korea

**Keywords:** Microtubules, Actin

## Abstract

Structural proteins such as actin and tubulin form the fundamental framework of the cytoskeleton and are essential for diverse cellular processes, including morphogenesis, intracellular transport and cell division. Maintaining precise intracellular levels is crucial for cellular homeostasis because both excess and deficiency can lead to cytotoxicity. Although transcriptional regulation establishes basal expression levels, recent studies have highlighted the crucial role of post-transcriptional and post-translational mechanisms in the fine-tuning of cytoskeletal protein abundance in response to dynamic cellular demands. Actin and tubulin use distinct autoregulatory strategies. Tubulin mRNA undergoes cotranslational decay, mediated by TTC5 and tightly regulated by the CARM1–PI3KC2α axis, linking ribosome-associated quality control with post-translational modifications. Conversely, actin regulation involves mRNA localization via ZBP1 and spatially restricted translation, coupled with a G-actin–MRTF/SRF transcriptional feedback loop. In addition, the ubiquitin–proteasome system modulates cytoskeletal protein turnover and fine-tunes microtubule dynamics. The dysregulation of these pathways has been implicated in various human diseases, including tubulinopathies, cancer and myopathies. In this Review, we summarize the multilayered regulatory networks that control actin and tubulin abundance, highlight recent advances in autoregulatory circuits and their disease relevance, and discuss future research directions for the therapeutic targeting of cytoskeletal proteostasis.

## Fine-tuning actin and tubulin for cellular homeostasis

Mammalian cells rely on structures called the cytoskeleton, which includes actin filaments and microtubules, to maintain their shape and function. This study explores how cells regulate the levels of actin and tubulin, the building blocks of these structures. When these proteins are not balanced, it can lead to cell damage. The authors review how cells control these proteins through various mechanisms. The study highlights that cells use transcriptional regulation and post-transcriptional mechanisms to manage protein levels. For example, when there is too much free tubulin, a feedback loop reduces its production by degrading its mRNA. Similarly, actin levels are controlled by feedback mechanisms that adjust mRNA stability based on the amount of unpolymerized actin. The findings emphasize the importance of these regulatory systems in maintaining cellular health.

This summary was initially drafted using artificial intelligence, then revised and fact-checked by the author.

## Introduction

Mammalian cells are supported by three major types of cytoskeletal structures: actin filaments; microtubules composed of α- and β-tubulin heterodimers; and intermediate filaments, such as keratin, vimentin and desmin^1^. These cytoskeletal components provide structural integrity, maintain the cell shape and facilitate intracellular transport and motility^2,3^. Actin and tubulin are the most abundantly expressed proteins in eukaryotic cells^3^. These high levels of expression present a substantial regulatory challenge: insufficient levels impair fundamental cellular functions, while excessive accumulation disrupts cytoskeletal dynamics, impairs organelle function, promotes protein aggregation and activates stress response pathways, ultimately leading to cellular toxicity^4–6^. To maintain cytoskeletal homeostasis, cells use multilayered regulatory systems capable of precisely and flexibly controlling the expression and activity of actin and tubulin^3,5,7^.

Transcriptional regulation provides a foundational framework for establishing the basal expression levels of cytoskeletal proteins in a cell type- and developmental stage-specific manner^8–10^. However, cellular demand for these proteins is often dynamic and fluctuates rapidly in response to environmental and physiological cues. As such, transcriptional control alone is insufficient to meet the immediate and context-specific requirements for cytoskeletal remodeling. Several studies have highlighted the essential roles of post-transcriptional and post-translational mechanisms in fine-tuning the abundance of cytoskeletal proteins with temporal precision^11–15^. A well-characterized example of such regulation is autoregulation, which is a feedback loop that is responsive to the levels of unpolymerized cytoskeletal monomers^16,17^. Elevated levels of monomeric G-actin inhibit further synthesis of the actin protein, thereby preventing excess accumulation^16,18,19^. Autoregulation of tubulin is mediated by a cotranslational feedback mechanism that directly senses free tubulin levels. When excess free tubulin binds to nascent tubulin chains emerging from the ribosome, it induces ribosome stalling and promotes selective degradation of the translated mRNA^17,20–22^. In addition to regulating mRNA levels, cytoskeletal protein abundance is modulated by the ubiquitin–proteasome system (UPS)^23^. For instance, γ-actin is targeted for degradation by the E3 ubiquitin ligase, tripartite motif-containing protein 3 (TRIM3)^24^, while α- and β-tubulin are regulated by the E3 ligase Parkin^25^. Together, these post-transcriptional and post-translational mechanisms constitute a highly responsive and integrated regulatory system that adjusts cytoskeletal protein levels in accordance with basal requirements and dynamic functional demands. These regulatory layers act as active guardians of cellular structure and homeostasis, rather than serving as passive outputs of gene expression.

In this Review, we provide an integrated overview of the molecular mechanisms that regulate the expression and turnover of highly expressed cytoskeletal proteins, with particular focus on actin and tubulin. Beginning with transcriptional regulation, we explored the subsequent layers of control, including mRNA stability, translational regulation and proteasomal degradation. We conclude by discussing the emerging feedback circuits and their physiological and pathological implications.

## Transcriptional regulation of cytoskeletal protein genes

Transcriptional regulation provides an essential framework for controlling cytoskeletal protein abundance, enabling cells to adjust actin and tubulin gene expression in response to developmental, differentiation and environmental factors. Although transcriptional control is not solely responsible for maintaining precise protein levels, it also underpins higher-order regulatory mechanisms.

## Gene family organization

**Fig. 1:**
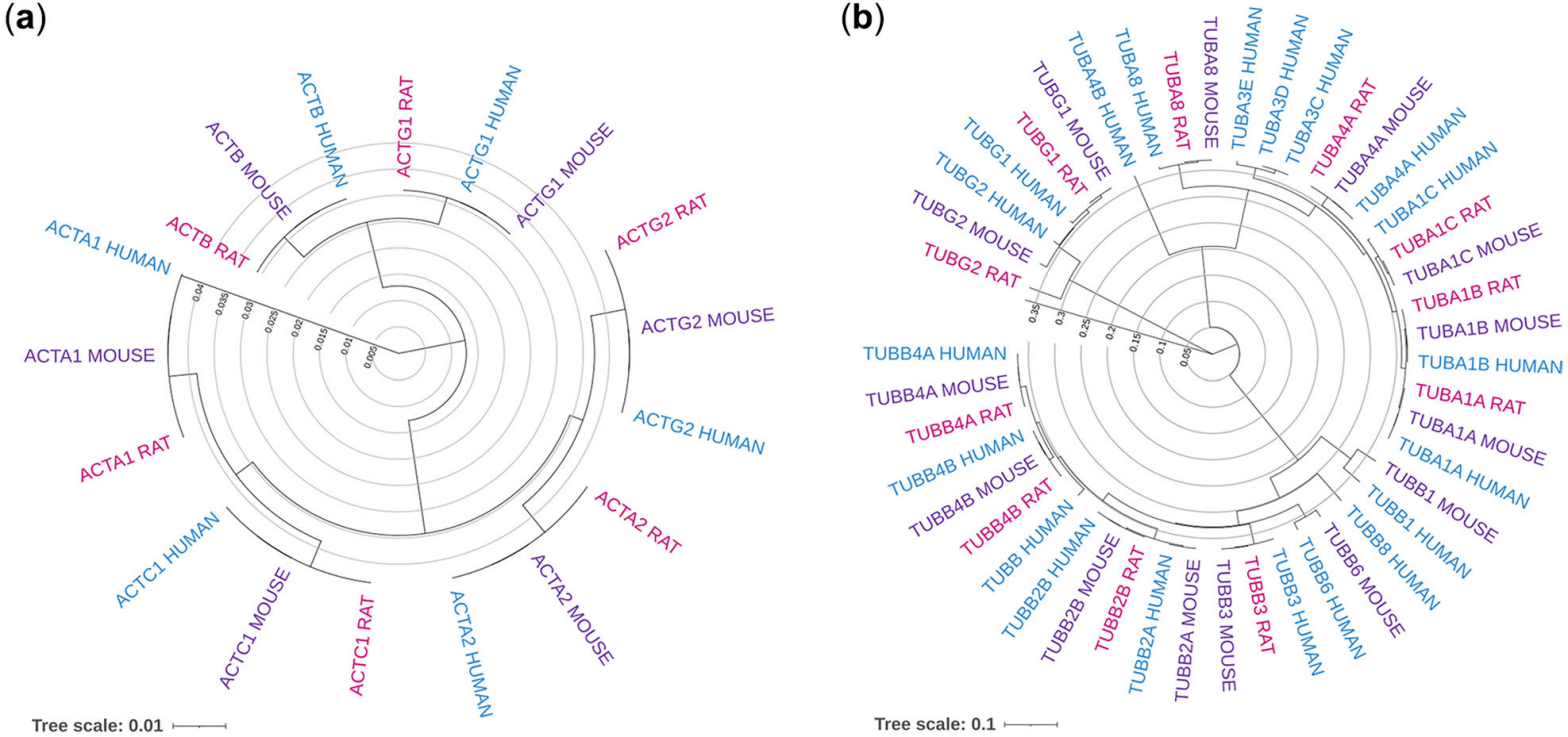
Phylogenetic analysis of actin isoforms and tubulin isotypes in humans, mice and rats. **a** Guide tree of actin isoforms from humans, mice and rats. The analysis includes six actin isoforms from each species: *ACTA1*, *ACTA2*, *ACTB*, *ACTC1*, *ACTG1* and *ACTG2*. Isoforms from humans, mice and rats are color-coded in blue, purple and pink, respectively. **b** Guide tree of tubulin isotypes from humans, mice and rats. The analysis includes nine α-tubulin (*TUBA*) and nine β-tubulin (*TUBB*) isotypes, as well as γ-tubulin (*TUBG*) from each species. Isoforms from humans, mice and rats are shown in blue, purple and pink, respectively. Guide trees were generated using neighbor-joining methods, and tree scales are indicated at the bottom of each panel.

In higher eukaryotes, actin and tubulin are encoded by evolutionarily conserved multigene families (Fig. 1). The human genome contains six actin isoforms with distinct and sometimes overlapping tissue-specific expression patterns^26^. Some are muscle specific, whereas others, such as *ACTB* and *ACTG1*, are ubiquitously expressed^27–29^. Knockout studies in mice have demonstrated that individual isoforms have specialized and nonredundant functions^27,28^. Actin promoters contain conserved *cis*-regulatory motifs (TATA box, CArG box and CCAAT box) that enable both basal- and tissue-specific transcription via general and specialized transcription factors^30–33^. For instance, β-actin expression is broadly maintained but can be modulated by serum response factor (SRF) and NF-Y^34,35^, while muscle-specific isoforms are regulated by muscle-specific regulatory factors such as MEF-2^36^. Post-transcriptional mechanisms, including mRNA stability and translation efficiency, further refine isoform abundance^27,28^. Similarly, nine isotypes each of α- and β-tubulin exist in humans, with unique tissue expression profiles^10,37^. Tubulin genes are mainly transcribed by general transcription factors to sustain cytoskeletal homeostasis; however, certain isotypes are also regulated by specific transcription factors and epigenetic modifications depending on the cellular context. For example, *TUBB3* is controlled by factors, such as AP-1, SP1, HREs and AREs and its expression is modulated by DNA methylation and histone modifications, particularly in cancer^38–40^. These layers of regulation ensure cell type- and context-specific expression of cytoskeletal proteins^41–44^.

## Tissue-specific and signal-dependent expression

**Fig. 2:**
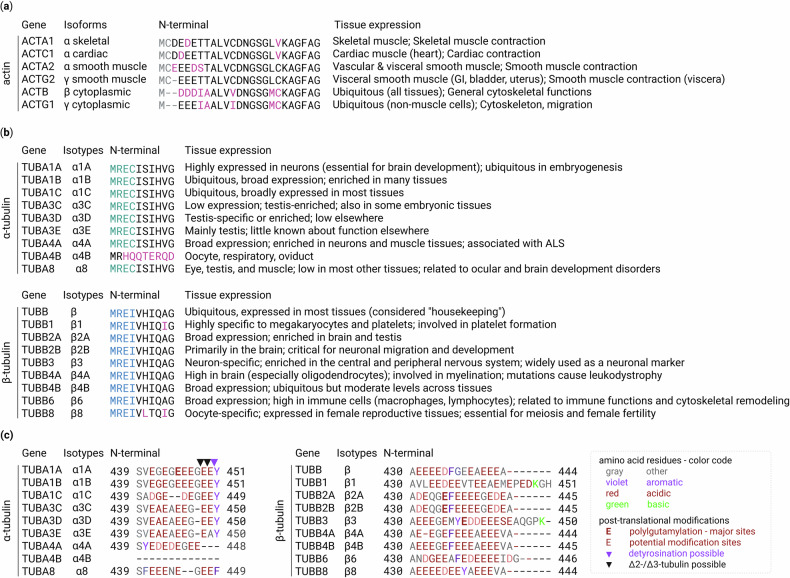
Actin isoforms and tubulin isotypes in humans. **a**,**b** A summary of the six human actin isoforms (**a**) and nine α-tubulin and nine β-tubulin isotypes (**b**). In **a**, the N-terminal sequences of actin isoforms are displayed, with sequence differences highlighted in pink. The gray regions at the N terminus indicate segments that are proteolytically removed and subsequently acetylated. In the case of β-actin, the processed N-terminus can also be arginylated. In **b** the N-terminal sequences of α-tubulin and β-tubulin isotypes are highly conserved, except for tubulin α4B, which shows some divergence. Sequence variations are highlighted in pink. Notably, the MREC motif (green) in α-tubulin and the MREI motif (blue) in β-tubulin are highly conserved across isotypes; these motifs are essential for autoregulation as they mediate interaction with TTC5 (Fig. 3). **c** Diversity of C-terminal tails and PTMs in human tubulin isotypes. Amino acids are color-coded according to their biochemical properties. Experimentally validated polyglutamylation sites are highlighted in bold, while all glutamate residues are shown as potential modification sites. Documented C-terminal truncations of α-tubulins are marked with ‘▼’.

Cytoskeletal gene expression is dynamically regulated during development and exhibits a clear tissue specificity^45^. Among the actin isoforms, *ACTA1*, *ACTA2*, *ACTC1* and *ACTG2* are muscle specific, whereas *ACTB* and *ACTG1* are ubiquitous^26^ (Fig. 2a). During early cardiac development, skeletal and cardiac α-actin are both expressed, with skeletal α-actin initially being more abundant. As the heart matures, cardiac α-actin expression increases and becomes the predominant isoform in the adult myocardium^46^. This isoform switch is essential for cardiac morphogenesis and function. Consistent with this, knockout studies in mice have highlighted the essential roles of specific isoforms during development, with *Actg1* deletion causing developmental abnormalities^47^ and *Actb* loss resulting in embryonic lethality^48^. Tubulin isotypes showed similar diversity; some were broadly expressed, whereas others displayed strong cell type specificity (Fig. 2b). For example, *TUBA1A* is strongly enriched in developing and post-mitotic neurons, whereas other α-tubulin isotypes (for example, *TUBA1B* and *TUBA1C*) exhibit broader, more ubiquitous expression. By contrast, *TUBB3* is widely expressed in neurons throughout both the central and peripheral nervous systems^49–51^. Mutations in *TUBA1A* are associated with severe brain malformations^52,53^, highlighting the importance of isotype-specific expression during neurodevelopment. The functional diversity of tubulin isotypes is underpinned by their structural features: while the N-terminal region is highly conserved, typically containing MREI or MREC motifs (Fig. 2b), the C-terminal region is markedly diverse. The latter not only serves as a hotspot for post-translational modifications (PTMs) but also provides a critical interface for interactions with microtubule-associated proteins (MAPs) and motor proteins (Fig. 2c). Although the mechanisms dictating isotype choice remain unclear, transcriptional regulation remains central. Meanwhile, cytoskeletal gene expression is rapidly modulated by external signals such as serum, growth factors (for example, EGF and PDGF) and mechanical stimuli, which drive changes in cell morphology, adhesion and motility^54–56^. A classic example is the activation of actin genes by SRF, which binds to CArG boxes in actin promoters.SRF activity is regulated by Rho-family GTPases and cooperates with myocardin-related transcription factors (MRTFs), whose nuclear localization is controlled by G-actin levels, thereby linking actin dynamics directly to transcription^57–59^. Tubulin gene regulation is more context dependent and involves diverse *cis*-regulatory elements in promoters, introns and untranslated regions (UTRs). For instance, elements, such as β2UE1 (spermatogenesis) and homeodomain/E-box motifs (neuronal α1-tubulin), mediate tissue- and stage-specific expression^60–63^. Additional regulations involve p53-binding intron regions (repression)^64^ and HIF-1α-binding 3′-flanking regions (induction under hypoxia)^65^. However, the complete regulatory landscape of tubulin gene expression remains unclear.

## Autoregulatory mechanisms of cytoskeletal proteins

Transcriptional regulation plays a pivotal role in establishing the basal expression levels of cytoskeletal proteins. However, they are often insufficient to accommodate rapid and context-specific fluctuations in protein demand. To achieve precise control of cytoskeletal protein abundance, mammalian cells use post-transcriptional regulatory strategies that modulate mRNA stability, degradation and subcellular localization. Among these, autoregulation is one of the most well-characterized mechanisms. This exemplifies the sophisticated feedback systems that operate at the mRNA levels to maintain cytoskeletal proteostasis. This mechanism underscores the extent to which cells have evolved multilayered and finely tuned regulatory networks to ensure dynamic and homeostatic control of highly abundant structural proteins.

## Cotranslational tubulin mRNA decay

The autoregulatory feedback mechanism that governs tubulin mRNA stability was first described in the 1980s^20^. A key observation was that, under conditions of excess free tubulin, unspliced pre-mRNAs remained stable, whereas mature tubulin mRNAs underwent selective degradation. This finding suggests that mRNA decay is tightly coupled with translation; that is, degradation is triggered specifically when ribosomes engage with mature transcripts^17,20–22^. However, the molecular sensors and effectors mediating this feedback loop have remained unidentified for decades. A major conceptual breakthrough occurred in 2020 when the Hegde group uncovered a tetratricopeptide repeat domain 5 (TTC5)-dependent regulatory pathway that directly links nascent tubulin synthesis to cotranslational mRNA decay^66^. TTC5 recognizes the conserved N-terminal motif of nascent α- and β-tubulin chains as they emerge from the ribosome. Upon binding, TTC5 recruits the adaptor protein SCAPER (S-Phase Cyclin A Associated Protein in the Endoplasmic Reticulum), the deadenylase complex CCR4–NOT (Carbon Catabolite Repression—Negative On TATA-less) and the substrate-recognition module CNOT11 (CCR4–NOT transcription complex subunit 11). This multicomponent complex facilitates selective degradation of the associated tubulin mRNA, thereby suppressing further tubulin synthesis^66,67^.

**Fig. 3:**
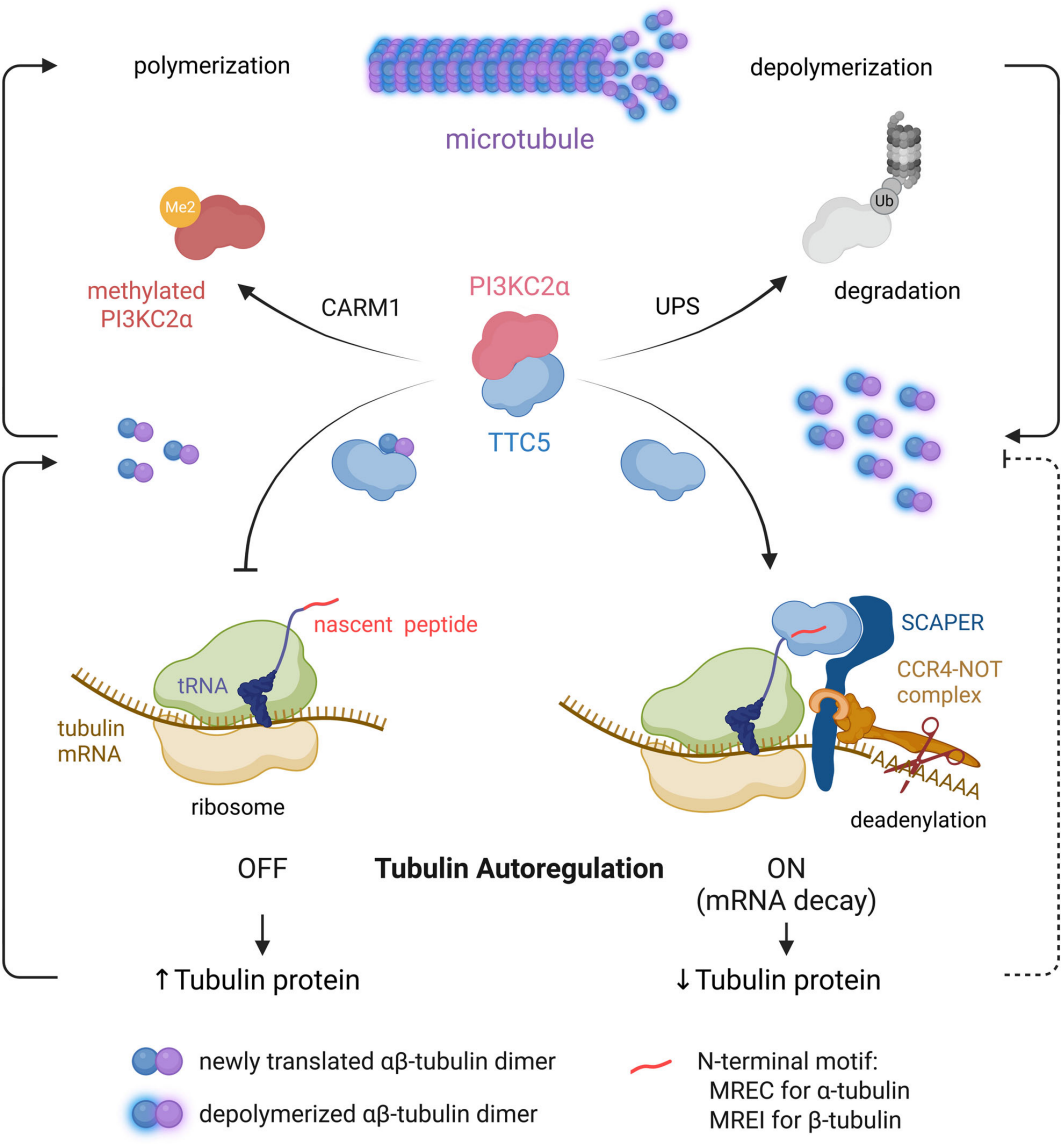
Mechanistic model of tubulin autoregulation via the CARM1–PI3KC2α–TTC5 signaling axis. PI3KC2α acts as a key regulatory factor in the tubulin autoregulatory pathway by binding to and sequestering TTC5, thereby suppressing TTC5-mediated cotranslational decay of tubulin mRNA. The release of TTC5 can be triggered via two distinct mechanisms: first, proteasomal degradation of PI3KC2α reduces its cellular abundance, resulting in the liberation of TTC5 (right); second, CARM1-mediated methylation of PI3KC2α diminishes its binding affinity for TTC5, thereby facilitating TTC5 release (left). Unmethylated PI3KC2α is inherently unstable and undergoes rapid degradation through the UPS, leading to a decrease in PI3KC2α levels and an accumulation of unpolymerized tubulin in the cytosol. Under these conditions, freed TTC5 associates with the MREC or MREI motif located at the N terminus of nascent tubulin chains emerging from ribosomes, thereby initiating cotranslational mRNA decay and activating the tubulin autoregulatory circuit. Conversely, when PI3KC2α is methylated by CARM1, it promotes microtubule polymerization and stabilizes microtubule networks, which in turn reduces the cytosolic pool of unpolymerized tubulin. In this methylated state, even if TTC5 is released, the limited availability of free tubulin or newly translated tubulin dimers fails to effectively trigger TTC5-mediated mRNA decay, thereby maintaining suppression of the autoregulatory pathway.

Despite these advances, the upstream regulatory mechanisms that control TTC5 activation remain poorly understood. Recent work by the Kim group has proposed a novel coactivator associated arginine methyltransferase 1 (CARM1)–phosphatidylinositol 3-kinase type 2α (PI3KC2α)–TTC5 signaling axis that offers insight into this regulatory hierarchy^68^ (Fig. 3). In its unmethylated form, PI3KC2α, a class II phosphatidylinositol 3-kinase previously implicated in endosomal trafficking and membrane remodeling^69^, binds to TTC5 and inhibits its interaction with ribosome–nascent chain complexes. In this context, PI3KC2α functions as a negative regulator of tubulin autoregulation by sequestering TTC5 under steady-state conditions. Specifically, two mechanistically distinct modes of PI3KC2α regulation contribute to the control of tubulin autoregulation: one governs TTC5 activity and the other alters microtubule dynamics through arginine methylation. First, when CARM1-mediated methylation of PI3KC2α is reduced, it becomes less stable, leading to the release of TTC5. This, in turn, enables TTC5 to associate with ribosome–nascent chain complexes and initiate cotranslational mRNA decay^68^. This mechanism underscores the role of PI3KC2α as a gatekeeper of TTC5 activity and highlights the importance of proteostasis in the dynamic regulation of cytoskeletal protein synthesis. Second, methylation of PI3KC2α at arginine 175 by CARM1 promotes microtubule polymerization and stability, thereby decreasing the intracellular pool of unpolymerized tubulin and indirectly suppressing activation of the autoregulatory machinery^70^. Notably, this represents a previously unrecognized form of post-transcriptional control in which protein arginine methylation regulates mRNA stability via the modulation of an upstream regulatory scaffold.

However, this model also raises a fundamental mechanistic question: If methylated PI3KC2α no longer binds to TTC5, why does TTC5 not activate the autoregulatory response? Although methylation appears to reduce the affinity between PI3KC2α and TTC5, it is unclear whether this dissociation alone is sufficient for TTC5 activation. This ambiguity indicated the presence of additional regulatory layers. Notably, a recent study observed that newly synthesized αβ-tubulin heterodimers sequester TTC5 and prevent its activation, whereas αβ-dimers released from depolymerized microtubules lack this capacity^71^. This distinction implies that the biochemical origin of free tubulin, and not just its abundance, may influence TTC5 function. Moreover, differential PTMs on nascent versus recycled tubulin may serve as molecular signatures that guide TTC5 binding or activity. Clarifying these nuances is critical for achieving a more comprehensive understanding of how microtubule homeostasis is maintained through tightly coordinated layers of transcriptional and post-transcriptional regulation.

## Transcriptional regulation of actin and its localized translation

**Fig. 4:**
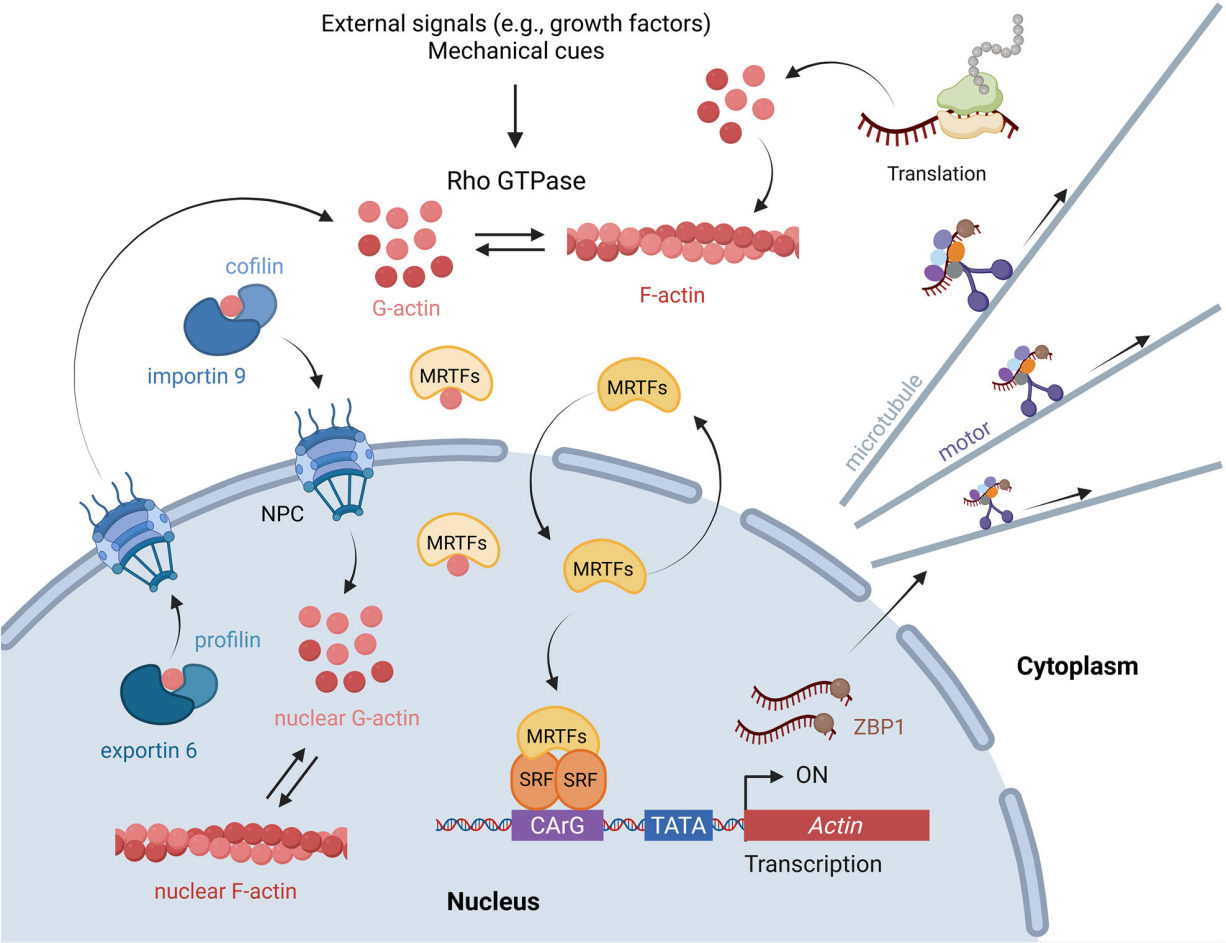
Autoregulatory mechanisms controlling intracellular actin level. This schematic illustrates the major autoregulatory mechanisms that control intracellular actin levels. At the transcriptional level, excess cytoplasmic G-actin binds to MRTF, sequestering it in the cytoplasm and thereby preventing its nuclear translocation. This interaction inhibits SRF-dependent transcription of actin genes, establishing a negative feedback loop that suppresses *ACTB* mRNA synthesis under conditions of elevated G-actin. At the post-transcriptional level, *ACTB* mRNA is recognized by ZBP1 and transported along microtubules to specific subcellular regions, where localized translation occurs. The newly synthesized actin monomers are then polymerized into filamentous F-actin, which can depolymerize back into G-actin, forming a feedback circuit that links transcriptional and translational control. Nuclear actin dynamics are further regulated by the nucleocytoplasmic shuttling of G-actin mediated by importin-9 and cofilin for nuclear import, and exportin-6 and profilin for nuclear export. Within the nucleus, G-actin binds MRTFs and inhibits SRF-dependent actin gene transcription. In response to specific stimuli, nuclear G-actin can also polymerize into F-actin, which contributes to nuclear functions such as chromatin accessibility, transcription and DNA damage repair. These multilayered regulatory mechanisms enable precise spatiotemporal control of actin expression in response to cellular demands. NPC, nuclear pore complex.

The concept of the autoregulation of actin expression was first proposed in the 1990s^72^. Experimental studies have shown that total cellular actin protein levels remain remarkably stable, even when actin mRNA levels fluctuate considerably^73,74^. This discrepancy suggests the presence of a compensatory feedback mechanism that maintains actin proteostasis at the post-transcriptional level^75^. A key finding supporting this hypothesis is that the intracellular concentration of monomeric G-actin regulates the stability of actin mRNA. When the cytoskeleton is pharmacologically disrupted to increase G-actin levels, actin mRNA levels decrease, indicating that the polymerization state of actin directly influences gene expression^18,19,76^. This establishes the existence of a feedback mechanism that links actin dynamics to mRNA stability. The molecular basis for this feedback was elucidated in the early 2000s, when researchers identified a specific nucleotide sequence—referred to as the zipcode—within the 3′-UTR of *ACTB* mRNA^77,78^. This zipcode is recognized by the RNA-binding protein zipcode-binding protein 1 (ZBP1; also known as IGF2BP1), which binds to mRNA and directs it along the microtubules toward the leading edge of migrating cells^79–81^. At this subcellular location, *ACTB* mRNA undergoes localized translation, thereby enabling spatial control of actin synthesis^81^. This mechanism demonstrates that mRNA localization and translational regulation are closely coupled and play crucial roles in coordinating cytoskeletal dynamics with cell polarity and motility (Fig. 4).

In addition to post-transcriptional control, actin expression is regulated at the transcriptional level through a feedback circuit involving the MRTF–SRF transcriptional complex^57^. G-actin binds to the RPEL motifs in MRTF-A, sequestering it in the cytoplasm and preventing its entry into the nucleus^57,82,83^. Because actin genes are direct transcriptional targets of SRF^84^, this interaction creates a classical negative feedback loop^85^. Specifically, an increase in G-actin leads to a greater sequestration of MRTF, which reduces SRF-mediated transcriptional activity, thereby decreasing the transcription of actin genes and ultimately reducing actin protein synthesis^34,86^. Interestingly, actin is not confined to the cytoplasm, but also exists in the nucleus, where it plays important regulatory roles^87–92^. Nuclear G-actin is actively maintained by importin-9 and cofilin. Unphosphorylated cofilin binds G-actin and forms a ternary complex with importin-9, facilitating cytoplasm-to-nucleus transport and supporting transcription and chromatin regulation, whereas its phosphorylation inhibits this import^93,94^. Actin export from the nucleus is mediated by exportin-6, which recognizes the profilin–actin heterodimer in a Ran-GTP-dependent manner^95^. Profilin promotes ADP–ATP exchange, enabling ATP-actin to be efficiently reused in cytoplasmic filament elongation^96^. This nucleocytoplasmic shuttling is dynamically regulated by the intracellular G-actin/F-actin ratio. Within the nucleus, G-actin reinforces the inhibition of transcriptional feedback by binding to MRTFs and attenuating their transcriptional activity^58^. In response to specific stimuli, such as signaling cues, stress or differentiation, nuclear G-actin polymerizes into F-actin^97^, relieving MRTF inhibition and triggering SRF-dependent transcription. This dynamic shift not only promotes actin gene expression but also exemplifies how the cytoskeletal state directly influences nuclear transcriptional programs.

This multilayered regulatory system allows the cells to finely tune the expression of actin or tubulin in response to the cytoskeletal status and mechanical demands of the environment. The key features of actin and tubulin autoregulation are summarized in Table 1. This feedback system ensures that actin is synthesized only when needed and prevents overaccumulation, which can disrupt cellular functions. Although notable mechanistic insights into actin autoregulation were obtained in the early 2000s, advances in this field have been relatively limited over the past two decades. By contrast, tubulin autoregulation has emerged as a highly dynamic and complex regulatory network that integrates multiple layers of control, including cotranslational mRNA surveillance, proteasome-mediated degradation and signaling pathways responsive to cellular stress and mitotic demand^66–68,70,71^. This disparity in regulatory complexity probably reflects intrinsic biological differences between actin and tubulin. Microtubules are central to highly dynamic processes, such as mitosis, vesicle trafficking and rapid structural remodeling, and thus require more rapid and precise control. Actin, although essential for many cellular processes, exhibits comparatively stable dynamics and is, therefore, subject to less complex autoregulatory control.

**Table 1:** The key features of actin and tubulin autoregulation.

Category	Actin	Tubulin
Definition	Regulation by free G-actin levels	Regulation by free αβ-tubulin dimer levels
Regulation mode	High G-actin levels regulate actin gene transcription via the MRTF/SRF pathway	CARM1–PI3KC2α–TTC5 axis controls tubulin mRNA stability
Mechanism	G-actin sequesters MRTFs, blocking SRF-dependent transcription	Nascent N-terminal motif recruits TTC5 to trigger cotranslational mRNA decay
Target Genes	β-Actin genes, other cytoplasmic actin genes	α- and β-tubulin genes (with MREC or MREI motif)
Physiological Role	Actin dynamics, motility, transcription, DNA repair, mechanotransduction	Microtubule dynamics, mitosis, transport
Features	Transcriptional feedback loop sensitive to G-actin levels; relatively slow due to transcriptional control	Rapid post-transcriptional regulation via mRNA decay, enabling swift adjustment of tubulin protein levels

## Protein turnover: the UPS

The maintenance of cytoskeletal homeostasis requires not only the timely and appropriate synthesis of actin and tubulin but also the selective degradation of excess, misfolded or unassembled proteins. To achieve this, cells have evolved sophisticated protein quality control mechanisms, of which the UPS plays a central role in targeting cytoskeletal proteins and their regulatory factors for proteolytic degradation^98,99^. In this pathway, target proteins are tagged with polyubiquitin chains by E3 ubiquitin ligases, which mark them for recognition and subsequent degradation by the 26S proteasome^100^. Unlike several signaling proteins or metabolic enzymes, actin and tubulin assemble into large dynamic polymers, and dysregulation of their polymerization dynamics can lead to cytoskeletal instability, proteotoxic stress and pathological protein aggregation. Therefore, the precise control of actin and tubulin, as well as their regulatory proteins, is essential for maintaining cytoskeletal integrity. Degradation of cytoskeletal regulators via the UPS is a critical component of this control, allowing the cell to fine-tune cytoskeletal assembly and remodeling in response to physiological demands. This degradation system broadly contributes to key cellular functions including migration, division and intracellular transport. Table 2 summarizes the ubiquitination sites and functional roles of actin, tubulin and their respective regulatory proteins. In this section, we discuss how ubiquitin-mediated degradation regulates actin and tubulin abundance, with particular emphasis on the turnover of associated regulatory proteins and their implications for cytoskeletal proteostasis.

**Table 2:** Summary of the ubiquitination of cytoskeleton-associated proteins.

**Substrate**	**E3 ubiquitin ligases**	**Ubiquitinated lysine residues**	**Function and characteristics**	**References**
Regulation of actin expression and dynamics				
γ-Actin	TRIM3	*K18	Cotranslational polyubiquitination and degradation	^24^
Cdc42	XIAP	K166	Proteasomal degradation	^107^
Cofilin	Cbl	*K19, K92, K112, K144, K164	K48-linked ubiquitination and degradation	^110^
	AIP4		K63-linked ubiquitination	
Formin mDia2	–	*K118	Proteasomal degradation	^192^
PAK1	–	–	Proteasomal degradation	^108^
Profilin 1	NEDD4	K70, K108	Proteasomal degradation	^193^
Rac1	HACE1	K147	Proteasomal degradation	^101^
	cIAP1, XIAP	K147	Proteasomal degradation	^102^
	SCF^FBXL19^	K166	Proteasomal degradation; depends on AKT-mediated S71 phosphorylation	^103^
RhoA	CUL3/BACURD	–	Proteasomal degradation	^104^
	SCF^FBXL19^	K135	Proteasomal degradation; ERK2-mediated phosphorylation-dependent mechanism	^105^
	Smurf1	K6, K7, K51	Proteasomal degradation; only the triple mutation (K6/7/51R) markedly reduces ubiquitination levels compared with WT or K6/7R; Smurf1 interacts with the dominant negative form of RhoA in vivo and with nucleotide-free or GDP-bound RhoA in vitro	^106^
ROCK2	APC/C^Cdh1^	–	Proteasomal degradation	^109^
Regulation of tubulin expression and dynamics				
α-Tubulin	MGRN1	*K60, K96, K112, K326	K6-linked polyubiquitination; promotes tubulin polymerization	^118,119^
	Parkin		Proteasomal degradation	^25^
β-Tubulin	Parkin	*K19, K58, K103, K252	Proteasomal degradation	^25^
γ-Tubulin	BRCA1/BARD1	K48, K344	Monoubiquitination; regulates centrosome number	^116,117^
	CUL1, CUL4A, CUL4B	*K48, K113	Proteasomal degradation; no impact of individual Lys-to-Arg mutations on protein levels	^112^
EB1	CRL3^KLHL21^	K100	Monoubiquitination; inhibits its interaction with microtubule, promoting microtubule disassembly	^194^
	Unknown CRL related to CSN (proposed)	–	Proteasomal degradation	^195^
EB3	SIAH-1	–	Proteasomal degradation	^196^
GCP2	CUL1, CUL4A, CUL4B	*K50, K92, K347, K541, K735	Proteasomal degradation; because some ubiquitination sites are located within their γ-tubulin binding region, stability of GCP2 and GCP3 depends on binding to γ-tubulin	^112^
GCP3	CUL1, CUL4A, CUL4B	*K379, K392, K482, K505, K511		^112^
Tau	CHIP	*K257, K259, K267, K274, K281, K290, K321, K343, K353, K375, K385	Proteasomal degradation; balance between CHIP and Hsp70 levels determine Tau levels; CHIP–Hsc70 complex ubiquitinates and degrades phosphorylated Tau in collaboration with UbcH5B	^113,115^
	Praja1	–	Proteasomal degradation	^114^
WDL3	COP1	–	Proteasomal degradation	^197^

*Putative ubiquitination sites from mass spectrometry data; not functionally validated.

## Ubiquitination of actin and its regulators

So far, studies on the ubiquitination of actin itself remain limited; however, polyubiquitination of γ-actin by TRIM3 has been reported^24^. Conversely, the ubiquitination of actin-regulatory proteins has been more extensively investigated, particularly those involved in actin cytoskeletal remodeling, such as Rho family GTPases (for example, Rac1, RhoA and Cdc42), their downstream effectors (for example, PAK1 and ROCK2) and actin-binding proteins, such as cofilin. Rac1, a key regulator of lamellipodia formation and cell motility, is ubiquitinated by multiple E3 ligases, including HACE1^101^, inhibitors of apoptosis (IAPs)^102^ and SCF^FBXL19^ (ref. ^103^), thereby modulating its stability and activity in dynamic cellular processes, such as migration. RhoA, which governs stress fiber formation and cell polarity, is ubiquitinated by several E3 ligases, such as CUL3/BACURD^104^, SCF^FBXL19^ (ref. ^105^) and Smurf1^106^, and has functional implications for cell motility, morphogenesis and cancer progression. Cdc42, another member of the Rho GTPase family involved in filopodia formation and polarity establishment, is targeted by XIAP for ubiquitination, and this process has been linked to the suppression of tumor metastasis^107^. The downstream effectors of Rho GTPases also undergo ubiquitin-mediated regulation. PAK1, a serine/threonine kinase activated by Rac1 and Cdc42, can be degraded in response to the anti-parasitic drug ivermectin, although the responsible E3 ligase remains unidentified. This degradation has been associated with the inhibition of cancer cell proliferation^108^. ROCK2, a RhoA effector involved in contractility and cytoskeletal tension, is ubiquitinated by the anaphase-promoting complex/cyclosome (APC/C), a process that is crucial for maintaining neuronal stability^109^. Cofilin, a key actin-depolymerizing factor, is phosphorylated at Ser3 and Tyr68 before ubiquitination and subsequent degradation. This modification cascade effectively attenuates its depolymerization activity, contributing to actin filament stabilization under specific physiological conditions^110^. Collectively, while direct ubiquitination of actin is relatively rare, targeted degradation of actin regulatory proteins through the UPS represents a central mechanism for the fine-tuned control of actin cytoskeletal dynamics and associated cellular functions.

## Ubiquitination of tubulin and its regulators

Tubulin ubiquitination was first reported in 1988 when it was observed that the microtubule network could associate with ubiquitin^111^. Initially, this phenomenon was primarily attributed to the presence of MAPs rather than tubulin itself^111^. However, subsequent studies revealed that α-, β- and γ-tubulin subunits can also undergo direct ubiquitination^112^. For example, the E3 ubiquitin ligase Parkin mediates the ubiquitination of αβ-tubulin heterodimers, promoting their degradation or recycling^25^. In addition, E3 ligase complexes containing CUL1, CUL4A and CUL4B have been shown to ubiquitinate γ-tubulin as well as γ-tubulin complex proteins (GCP2 and GCP3), which are essential for microtubule nucleation at the centrosome^112^. Among the best-characterized ubiquitinated MAPs is Tau. Tau, a neuronal microtubule-stabilizing protein implicated in neurodegenerative diseases, is regulated by several E3 ligases^113,114^. CHIP, in cooperation with HSP70, promotes Tau ubiquitination and degradation^113^, and the E4 ligase UBE4B extends polyubiquitin chains to enhance degradation efficiency^115^. In addition, Praja1 has been identified as a novel E3 ligase that ubiquitinates Tau, contributing to its proteasomal turnover^114^.

Although actin ubiquitination is largely associated with proteasomal degradation, tubulin ubiquitination can also have nonproteolytic regulatory functions. For example, the BRCA1 (breast cancer susceptibility protein 1)–BARD1 (BRCA1 associated RING domain 1) complex monoubiquitinates γ-tubulin, a modification implicated in the regulation of centrosome number^116,117^. Similarly, mahogunin ring finger 1 (MGRN1) catalyzes the K6-linked polyubiquitination of α-tubulin, which does not trigger degradation but contributes to microtubule stabilization and spindle positioning^118,119^. In summary, the UPS plays a crucial role in the maintenance of cytoskeletal homeostasis by regulating the abundance and turnover of tubulin and its associated proteins. This is achieved not only through degradation-oriented pathways but also via nondegradative ubiquitination that modulates microtubule dynamics. Continued investigation of these regulatory circuits is expected to advance our understanding of cytoskeletal remodeling under both physiological and pathological conditions.

## Implications for disease and therapeutic opportunities

Tight regulation of actin and tubulin abundance is essential for preserving cellular structure and function. Disruption of this homeostatic balance contributes to the pathogenesis of numerous diseases with distinct consequences depending on the specific cytoskeletal system affected^120,121^. Due to the divergent regulatory architectures governing actin and tubulin expression, including differences in feedback mechanisms, transcriptional control and post-transcriptional modulation, perturbations in homeostasis result in nonoverlapping disease phenotypes. Actin dysregulation has been predominantly implicated in skeletal and cardiac myopathies^122^, as well as angiogenesis-related pathologies^123^. Given the complexity and ubiquity of actin networks, the direct pharmacological targeting of actin is often infeasible or nonspecific. Consequently, therapeutic strategies have largely focused on modulating upstream regulatory pathways, such as the Rho family of GTPases and the MRTF–SRF transcriptional axis^124^, to restore actin-dependent functions in disease contexts^125,126^. Conversely, aberrations in tubulin expression or dynamics are closely associated with neurodegenerative disorders^127^, malignancies^128^ and resistance to chemotherapeutic agents^129^. Current therapeutic approaches frequently exploit the dynamic instability of tubulin using microtubule-targeting agents, such as taxanes and vinca alkaloids, which interfere with polymerization dynamics^130^. However, these treatments are often limited by their toxicity and acquired resistance^130,131^. A more nuanced understanding of tubulin regulation, particularly at the translational, autoregulatory and PTM levels, may enable the development of next-generation therapeutics that target specific regulatory nodes, minimize off-target effects and overcome resistance to treatment.

## Actin dysregulation and disease

Pathogenic mutations have been identified in all six human actin genes, each exhibiting distinct expression patterns and disease associations^27^. Among these, *ACTA1*, which is exclusively expressed in the skeletal muscle, harbors the greatest number of disease-causing variants. Mutations in *ACTA1* are the predominant genetic cause of nemaline myopathy, a congenital disorder characterized by muscle weakness and respiratory impairment due to the formation of nemaline bodies in the muscle fibers^132,133^*. ACTA2*, primarily expressed in smooth muscle, is the second most frequently mutated actin gene and is strongly linked to familial thoracic aortic aneurysm and dissection^134^. Mutations in *ACTC1*, which is predominantly expressed in cardiac muscles, are associated with both hypertrophic and dilated cardiomyopathies^135,136^, highlighting the essential role of actin in maintaining myocardial structure and function. Conversely, mutations in the ubiquitously expressed *ACTB* result in a broad spectrum of clinical manifestations, including developmental delays, cerebral malformations, defects in neuronal migration and progressive muscle atrophy, collectively referred to as Baraitser–Winter cerebrofrontofacial syndrome^137,138^. Although *ACTG1* is widely expressed, pathogenic mutations in this gene tend to result in more restricted phenotypes^139,140^. The least commonly mutated gene *ACTG2*, which is expressed specifically in smooth muscles, has been implicated in megacystis–microcolon–intestinal hypoperistalsis syndrome, a rare and severe congenital visceral myopathy^141,142^. Despite the growing number of reported pathogenic actin variants, the molecular consequences of most mutations remain poorly understood. Ongoing in vitro and in vivo studies are aimed at delineating how these mutations alter actin polymerization dynamics, protein–protein interactions and cytoskeletal integrity to drive disease pathology.

In addition to these inherited disorders, dysregulation of actin dynamics plays a pivotal role in cancer metastasis^143^, further underscoring the central importance of actin in human diseases. Unlike tubulin, whose total expression levels are often directly linked to metastasis^144^, the role of actin in cancer progression primarily involves changes in actin dynamics rather than alterations in total protein levels^145^. Key actin-binding proteins such as cofilin, the Arp2/3 complex, fascin, gelsolin and profilin regulate actin polymerization and turnover, driving processes such as lamellipodia and filopodia formation, which are essential for metastasis^146–148^. In addition, actin mRNA localization to the leading edge and isoform switching between β-actin and γ-actin contribute to metastatic behavior^149–151^. Thus, while the total actin abundance remains relatively stable, its spatial regulation and dynamic remodeling are central to the invasive and metastatic potential of cancer cells. Moreover, this dysregulation has been implicated in age-associated disorders and vascular pathologies, reflecting its critical roles in cell motility, adhesion, mechanotransduction and cell cycle progression^152^. Aberrant activation of the RhoA–ROCK signaling pathway contributes to endothelial dysfunction, vascular stiffening and thrombotic complications^153^. A recent study demonstrated that constitutively active RhoA mutants (G14V/Q63L) inhibit endothelial cell proliferation and migration^154^, underscoring the pathological relevance of hyperactive RhoA signaling and the therapeutic potential of its inhibition.

Several pharmacological strategies have emerged to modulate actin dynamics, either by targeting upstream regulators or by directly interfering with actin filament assembly. ROCK inhibitors such as Fasudil, Ripasudil and Y-27632 have demonstrated therapeutic efficacy in preclinical and clinical models of vascular disease, neurodegeneration and glaucoma^155,156^. In addition, small molecules that modulate the actin architecture through distinct mechanisms have been developed. These include Rho inhibitors (for example, C3 transferase^157^ and Rhosin^158^) and compounds that act directly on actin filaments. For instance, Cytochalasin D caps the barbed ends of F-actin, preventing polymerization^159^; Latrunculin A sequesters G-actin monomers, thereby blocking filament assembly^160^; and Jasplakinolide stabilizes and nucleates F-actin by binding to filamentous structures^161^. Although potent, these agents often elicit broad cytoskeletal disruption and exhibit cytotoxic effects, thereby limiting their clinical applicability. Thus, although pharmacological manipulation of actin dynamics holds considerable therapeutic promise, it remains technically challenging because of the ubiquitous and multifunctional nature of the actin cytoskeleton. Future advances in isoform-specific targeting, spatiotemporal delivery and context-dependent modulation of actin regulators may enable safe and precise correction of actin dysregulation in human diseases.

## Tubulin dysregulation and disease

Mutations in specific tubulin isotypes underlie a spectrum of neurodevelopmental disorders collectively referred to as tubulinopathies, which are characterized by malformations of cortical development, motor impairments, and epileptic seizures^162^. These conditions are most commonly associated with heterozygous missense mutations in *TUBA1A*^52,163^, *TUBB2B*^164^, *TUBB3*^51^, *TUBB4A*^165^ and *TUBG1*^166,167^. Among them, *TUBA1A* mutations, particularly at conserved residues such as R264, R402 and R422, are strongly associated with lissencephaly and central pachygyria. By contrast, mutations in *TUBB2B* (for example, A248, R380 and E421) and *TUBB3* (for example, E205 and E410) have been implicated in polymicrogyria-like cortical dysplasia and multifocal cortical malformations^168^. These residues are located on the external surface of the tubulin dimer and participate in interdimer and intradimer interactions, suggesting that such mutations impair proper folding, dimerization and microtubule dynamics^169,170^. Supporting this, recent studies have demonstrated that mutations in *TTC5* or *PIK3C2A*, which are key components of the tubulin autoregulatory pathway, also lead to neurodevelopmental abnormalities, including global developmental delay, ventricular enlargement and cerebral atrophy^171,172^, highlighting the critical role of post-transcriptional regulation of tubulin homeostasis during brain development.

Intriguingly, despite the identification of over 290 pathogenic variants across tubulin genes, no disease-associated mutations have been reported at the K40 residue of α-tubulin or within the C-terminal tails of α- and β-tubulins^173^. These regions are known hotspots of PTMs and mediate their interactions with MAPs^174^. In particular, the α-tubulin K40 residue, located on the luminal face of microtubules, serves as a hotspot for diverse PTMs with distinct functional consequences. K40 acetylation by α-tubulin acetyltransferase 1 (αTAT1) stabilizes long-lived microtubules and enhances resistance to mechanical stress, intracellular transport and neuronal migration^175,176^. By contrast, SETD2-dependent K40 trimethylation promotes mitotic fidelity and microtubule stability^177,178^, whereas the recently described K40 lactylation exhibits differential enrichment on soluble versus polymerized tubulin, potentially modulating microtubule dynamics^179,180^. These modifications, which can compete for the same residue, collectively fine-tune microtubule behavior, cellular motility and neuronal development. Importantly, in the broader context of the tubulin code, such residue-specific PTMs act in combination with isotype diversity and C-terminal tail modifications to generate a regulatory language that dictates microtubule dynamics and MAP/motor binding. Their remarkable evolutionary conservation probably reflects their strong functional constraints. In support of this, deletion of the C-terminal tyrosine of α-tubulin in mice results in severe brain malformations and perinatal lethality^181^, and β-tubulin lacking its C-terminal tail can still polymerize but generates microtubules with aberrant dynamics and altered mechanical properties^182^. These findings highlight the essential contributions of PTMs to microtubule integrity, spatiotemporal dynamics and cellular resilience, thereby forming a mechanistic bridge between genetic lesions and structural cytoskeletal disruption.

Although genetic mutations highlight the role of tubulin in development, dysregulated expression and aberrant PTMs of tubulin are also critically involved in cancer progression and metastasis. Tumor cells frequently exhibit altered isotype compositions, PTM signatures and microtubule behavior^183,184^. For instance, β3-tubulin is overexpressed in lung, ovarian, breast and gastric cancers and is associated with increased tumor aggressiveness and poor response to therapy^185–187^. Similarly, nuclear-localized β2-tubulin correlates with adverse clinical outcomes in multiple cancer types^188^. γ-Tubulin, classically associated with microtubule nucleation, is often upregulated and mislocalized to the nucleus in high-grade tumors such as glioblastoma multiforme^189^. PTM dysregulation also drives chromosomal instability, enhanced motility and chemoresistance. For example, loss of tubulin tyrosine ligase like 1 (TTLL11), a tubulin polyglutamylase, disrupts accurate chromosome segregation^190^, and elevated αTAT1-mediated acetylation is observed in paclitaxel-resistant cells, reducing drug-induced apoptosis^191^.

These findings collectively highlight tubulin not only as a structural component, but also as a key regulator of both tumor progression and metastasis through isoform-specific expression, PTMs, autoregulation and dynamic remodeling of the microtubule network. Consequently, targeting tubulin-modifying enzymes or modulating PTMs offers promising therapeutic avenues, particularly in overcoming the limitations of conventional tubulin-targeting agents, such as taxanes (for example, paclitaxel) and vinca alkaloids (for example, vincristine), which often face resistance and dose-limiting toxicities^130,131^.

## Concluding remarks and future perspectives

The precise regulation of cytoskeletal protein abundance is fundamental for maintaining cellular homeostasis, enabling cells to adapt dynamically to changing physiological demands. Among cytoskeletal proteins, actin and tubulin are particularly abundant and essential; however, they are regulated by distinct autoregulatory mechanisms that reflect their divergent biological roles and evolutionary pressures. Tubulin homeostasis is governed by a highly dynamic, multilayered autoregulatory system that integrates translational surveillance, proteostasis and signal-responsive feedback. The cotranslational mRNA decay pathway initiated by TTC5 upon sensing nascent tubulin chains ensures that excess free tubulin rapidly suppresses its own synthesis. This mechanism is further fine-tuned by upstream regulators, such as PI3KC2α and its arginine methylation by CARM1, linking cytoskeletal dynamics to broader signaling pathways. The evolutionary elaboration of such a responsive and tightly coupled system probably reflects the critical role of microtubules in fast and reversible processes, such as mitosis, intracellular transport and rapid morphological remodeling. These functions require the real-time adaptation of tubulin abundance to the structural and temporal demands of the cell, necessitating feedback systems with high temporal resolution and signal sensitivity. By contrast, actin autoregulation evolves through transcription-centric and spatially coordinated mechanisms. Although actin mRNA stability is influenced by the cellular G-actin pool, post-transcriptional feedback is limited in terms of scope and mechanistic complexity. Instead, actin expression is modulated through transcriptional feedback involving the MRTF–SRF pathway and the spatially restricted translation via zipcode-binding proteins. These systems appear to be sufficient to maintain actin levels in accordance with the relatively stable demand for cell shape maintenance, migration and adhesion. Unlike the rapid assembly–disassembly dynamics of microtubules, actin structures—particularly stress fibers and cortical actin—undergo slower remodeling, providing less immediate but more spatially regulated control.

Therefore, the evolutionary divergence of these autoregulatory strategies may reflect a tradeoff between spatial precision and temporal responsiveness, optimized for the distinct physiological roles of actin and tubulin. Tubulin regulation prioritizes speed and feedback sensitivity, while actin regulation emphasizes localization and structural integrity. However, several key questions remain to be addressed in future studies. For example, it remains unclear how various PTMs such as tubulin acetylation or actin phosphorylation modulate the activity and dynamics of their respective autoregulatory circuits. Another critical question is whether the distinct states between newly synthesized and depolymerized proteins act as molecular cues within these regulatory pathways. In the case of tubulin, maintaining an appropriate stoichiometric balance between α-tubulin and β-tubulin is essential, as these subunits form obligate heterodimers that constitute the fundamental building blocks of microtubules. Elucidating the molecular mechanisms and regulatory networks that control α/β-tubulin homeostasis represents a fundamental challenge and a key priority for future research. Furthermore, understanding how these autoregulatory pathways are dysregulated in pathological contexts, particularly in cancer and neurodevelopmental disorders, may reveal novel therapeutic targets and offer new opportunities for clinical interventions.

Future studies should aim to uncover additional layers of regulation, including the roles of RNA-binding proteins, alternative splicing, noncoding RNAs and organelle-specific degradation systems. Expanding our knowledge of cytoskeletal autoregulation will not only deepen our understanding of fundamental cell biology, but also inform strategies for modulating the cytoskeleton in clinical contexts where its balance is perturbed.
